# miR-424对非小细胞肺癌A549细胞生长和侵袭的影响及分子机制

**DOI:** 10.3779/j.issn.1009-3419.2016.09.02

**Published:** 2016-09-20

**Authors:** 宏敏 李, 海涛 兰, 明 张, 宁 安, 瑞莲 于, 阳科 何, 崇志 甘

**Affiliations:** 1 610072 成都，四川省医学科学院四川省人民医院肿瘤中心 Cancer Center, Medical Sciences Academy of Sichuan, Renming Hospital of Sichuan Province, Chengdu 610072, China; 2 610072 成都，四川省医学科学院四川省人民医院胸外科 Department of Thoracic Surgery, Medical Sciences Academy of Sichuan, Renming Hospital of Sichuan Province, Chengdu 610072, China

**Keywords:** 肺肿瘤, miR-424, 生长, 侵袭, Lung neoplasms, MiR-424, Growth, Invasion

## Abstract

**背景与目的:**

已有的研究表明miR-424可抑制肾癌细胞增殖，抑制宫颈鳞癌细胞的迁移和侵袭能力，而其对非小细胞肺癌（non-small cell lung cancer, NSCLC）细胞的影响目前尚无系统研究。本研究探讨miR-424对NSCLC A549细胞生长和侵袭迁移能力的影响并进一步研讨其分子机制。

**方法:**

应用CCK8检测过表达及抑制miR-424的表达对A549细胞增殖的影响。应用Transwell检测过表达及抑制miR-424的表达对A549细胞侵袭能力的影响。应用Western blot检测过表达及抑制miR-424的表达对A549细胞中MMP9和MMP2蛋白水平的影响。构建E2F6 3’UTR区的荧光素酶报告载体，验证miR-424对E2F6的靶向作用。采用Western blot检测过表达及抑制miR-424的表达后，A549细胞中E2F6的表达。

**结果:**

过表达miR-424后，A549的生长和侵袭能力显著降低。过表达miR-424后，A549细胞的MMP-2和MMP-9表达下降。荧光素酶活性检测表明miR-424能够抑制E2F6的荧光素酶活性。过表达miR-424后，细胞内E2F6的表达降低。

**结论:**

miR-424能够通过调控E2F6而抑制A549的生长和侵袭能力。

## Abstract (en)

**Background and objective:**

The inhibitory ability of miR-424 on the proliferation of renal carcinoma cell and the migration and invasion of cancer cells has been widely explored and demonstrated. However, the effects of miR-424 on non-small cell lung cancer (NSCLC) have not been systematically examined. In this study, detected the growth and invasion effect of miR-424 in NSCLC A549 cell. The migration and molecular mechanism of this cell are also detected.

**Methods:**

NSCLC A549 cell was transfected with miR-424 and its inhibitor. After transfection, the proliferation ability of A549 cell was detectedby CCK8 assay. Then, the migration ability in A549 cell was detected by migration assays. Furthermore, the expression level of MMP2 and MMP9 in A549 was detected by Western blot and immune fluorescence. The 3'UTR of E2F6 was cloned into luciferase reporter vector and its enzymatic activitywas detected to verify whether miR-424 can target E2F6. The expression level of E2F6 in a549 cell after transfecing with miR-424 was detected by Western blot.

**Results:**

After transfection of miR-424, the proliferation and migration abilities were remarkably decreased and the expression level of MMP-2 and MMP-9 were down-regulated in A549. Moreover, MiR-424 inhibited the enzymatic activity of luviferase reporter vector of E2F6. Specifically, the expression level of E2F6 was down-regulated in A549.

**Conclusion:**

miR-424 can inhibit the proliferation and migration abilities of A549 by negatively regulating the expression of E2F6.

肺癌是近年来全球范围内发病率和死亡率最高的恶性肿瘤之一，其发病率和死亡率逐年上升^[1, 2]^。肺癌中约85%为非小细胞肺癌（non-small cell lung cancer, NSCLC）^[1]^，其发病率在肺癌中居首位^[2]^，患者5年内生存率不足15%^[2]^。研究NSCLC的发生发展机制对肿瘤的诊断及预后有重要意义^[3-6]^。MicroRNA（miRNA）是在真核生物中发现的一类高度保守的非编码小RNA，在细胞生命活动的调控以及多种肿瘤的发生发展中发挥重要作用^[2]^。研究表明，miR-424可通过靶定WEE1抑制肾癌细胞增殖^[2]^，miR-424能够抑制宫颈癌细胞细胞迁移和侵袭能力^[1]^，提示miR-424发挥着抑癌基因的作用，目前关于miR-424在NSCLC中的作用尚无相关研究。而有文章^[15]^指出，E2F6的低表达可能与宫颈鳞癌细胞的发生、发展、浸润有关。本研究旨在探讨miR-424是否影响NSCLC的生长和侵袭能力，并初步探索其分子机制是否与E2F6有一定关系。

## 1 材料与方法

### 1.1 材料

1640培养基、胎牛血清、Opti-MEM购自Gibco公司。免疫印迹化学发光系统购自Syngene公司。MMP-2和MMP-9的抗体购自Abcam公司。miR-424 mimics、miR-424 inhibitor购自Biomics公司，Trizol、逆转录试剂盒、PCR引物、Lipofectamine RNAi MAX购自美国Invitrogen公司。Transwell培养板购自Corning公司；Matrigel购自BD公司；X-tremeGENE购自罗氏公司。CCK8试剂购自日本同仁化学研究所。双荧光素酶检测试剂盒购自Promega公司。肺癌细胞株A549由本实验室保存。

### 1.2 方法

#### 1.2.1 细胞培养

肺癌细胞A549培养于含10%FBS的1640培养基中，培养液含青霉素/链霉素100 U/mL将细胞置于37 ℃、5%CO_2_培养箱中培养。

#### 1.2.2 细胞转染

取对数生长期的A549细胞，5×10^5^个/孔细胞接种于6孔板中，37 ℃、5%CO_2_培养24 h后用Lipo2000分别转染miR-424 mimics、miR-424 inhibitor以及miRNA con于细胞中，每组设三个复孔。

#### 1.2.3 实时定量RT-PCR检测miR-424的表达

用Trizol法提取转染48 h后的A549细胞总RNA，反转后进行RT-PCR反应，反应条件为：95 ℃，3 min；95 ℃，10 s；60 ℃，30 s，40个循环。以U6为参照，进行分析。

#### 1.2.4 CCK8检测细胞增殖

将转染48 h后的A549细胞培养基弃掉，每孔加入100 μL CCK8试剂和完全1640培养基的混合液（1:1），37 ℃、5%CO_2_培养箱孵育3 h后，测定490 nm波长处的吸光度值。

#### 1.2.5 Transwell检测细胞的侵袭能力

将铺有基质胶的孔径8 μm的Transwell培养板置于37 ℃预热，将转染24 h的细胞消化，用无血清1640培养基洗涤两次，调整细胞密度为1×10^5^个/mL，250 μL/孔，接种到Transwell上室中，下室加入800 μL含15%血清的1640培养液。37 ℃培养20 h，取出Transwell培养板，PBS洗涤后，在4%甲醛固定10 min，用1%结晶紫进行染色，显微镜下观察、照相。随机选取55个高倍视野（×200）计数每个视野下发生侵袭的细胞。

#### 1.2.6 Western blot检测蛋白表达

取对数生长期的A549细胞，以1×10^7^个/mL接种于10 cm^2^培养皿中，1 mL/盘，分别转染miR-424 mimics与miR-424 inhibitor，48 h后裂解细胞，收集蛋白进行Western blot检测。检测方法：将细胞用PBS洗涤后用加入预冷500 μL PBS，用细胞刮挂下细胞，1, 000 rpm，4 ℃离心10 min，向沉淀中加入300 μL细胞裂解液裂解细胞提取总蛋白。SDS-PAGE凝胶电泳分离，恒流300 mA转移至PVDF膜。5%脱脂牛奶封闭2 h后，加入一抗，4 ℃孵育过夜。次日PBST洗膜，二抗室温孵育2 h，PBST洗膜。用化学发光法显色，凝胶成像系统采集成像。

#### 1.2.7 荧光素酶报告载体的构建

在Targetscan中找出可能与miR-424作用的E2F6的3' UTR序列，用Primer 5.0软件分别设计合成野生型与突变型E2F6的3' UTR的PCR引物。分别以A549细胞cDNA为模板进行PCR扩增。PCR产物纯化回收后与连接到pmirGLO vector载体上，测序鉴定，得到野生型与突变型荧光素酶报告载体：pmirGLO/E2F6-3’UTR与pmirGLO/E2F6-3’UTR mut。

#### 1.2.8 双荧光素酶报告基因检测

将构建好的mirGLO/E2F6-3’UTR与pmirGLO/E2F6-3’UTR mut报告载体分别与miR-424 mimics、miR-424 inhibitor以及miRNA con，用X-treme GENE转染试剂共转染到A549细胞中，48 h后裂解细胞，用双荧光素酶检测试剂盒检测荧光素酶活性。测定实验组（A1）和海肾荧光素酶表达载体（A2）的活性，计算A1/A2比值表示相对荧光素酶活性。

#### 1.2.9 统计学分析

应用SPSS 12.0软件进行统计学处理，数据采用Mean±SD描述，不同组间数据比较应用*t*检验，以*P* < 0.05为差异有统计学意义。

## 2 结果

### 2.1 miR-424对NSCLC细胞增殖能力的影响

为确定miR-424对NSCLC细胞增殖的影响，将miR-424 mimics、miR-424 inhibitor以及miRNA con分别转染A549细胞。RT-PCR检测miR-424的表达变化，验证miR-424转染效果如图 1A所示；CCK8法检测细胞增殖情况。结果显示：与对照组相比，转染miR-424后，A549细胞增殖速率降低，而转染miR-424 inhibitor组A549细胞增殖速率提高，说明miR-424能够抑制NSCLC细胞的增殖（图 1B）。

**1:**
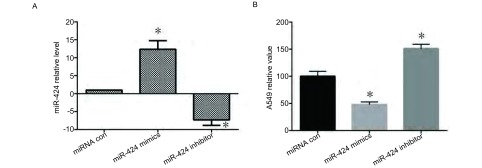
miR-424对A549增殖能力的影响。A：miR-424转染效果；B：CCK8法检测A549细胞增殖速率。^*^：与对照组相比，*P* < 0.05。 The effect of miR-424 on proliferation abilities in A549. A: Results of miR-424 transfection; B: The proliferation rate of A549 by CCK8. ^*^: compared with the control, *P* < 0.05.

### 2.2 miR-424对NSCLC细胞侵袭能力的影响

Transwell细胞侵袭实验结果如图 2A、图 2B，结果显示，与miRNA con组相比，转染miR-424组的A549细胞穿膜数降低（107.6±12.1 *vs* 45.7±6.6），而抑制miR-424表达后细胞的穿膜数提高（132.8±14.2）。说明miR-424能够抑制A549细胞的侵袭能力。

**2:**
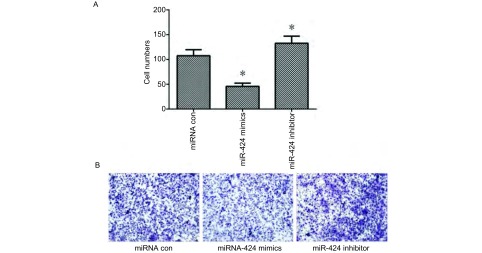
miR-424对A549细胞侵袭能力的影响。A：显微镜计数结果；B：结晶紫染色法检测miR-424对A549侵袭能力的影响（×200）。^*^：与对照组相比，*P* < 0.05 The migration abilities of miR-424 on A549. A: Results of microscope counting; B: The effects of miR-424 on migration abilities of A549 by crystal violet staining. ^*^: compared with the control, *P* < 0.05.

### 2.3 miR-424对NSCLC细胞中侵袭相关蛋白表达的影响

为探究miR-424对NSCLC细胞侵袭能力影响的分子机制，本研究检测了各转染组细胞中侵袭相关蛋白MMP-2和MMP-9的表达水平。结果显示，过表达miR-424后，两种蛋白表达水平降低，而抑制miR-424的表达后，两种蛋白表达水平提高，结果如图 3所示。

**3:**
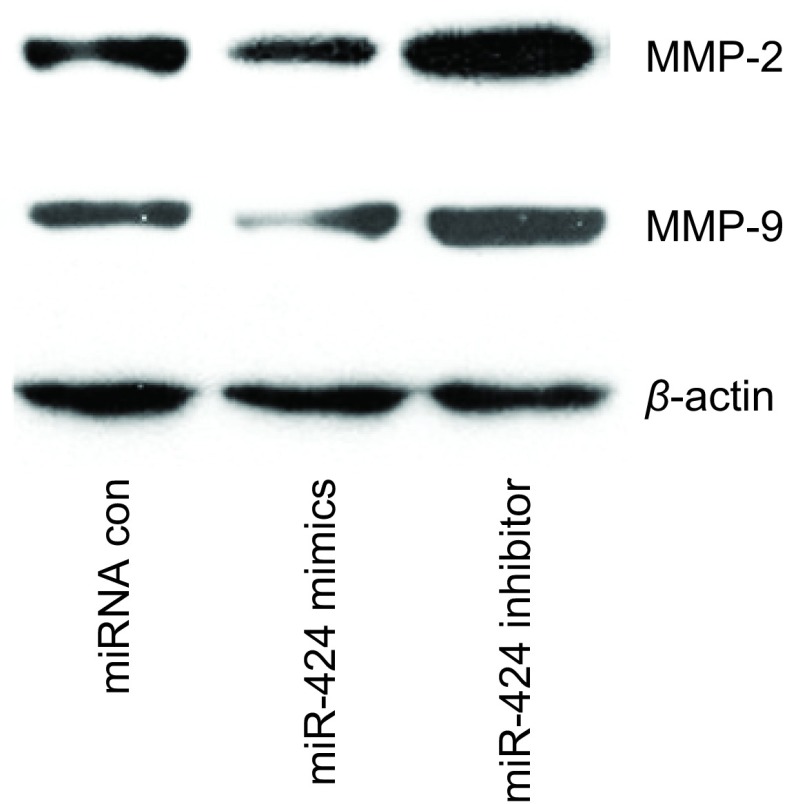
miR-424对A549中MMP-2和MMP-9表达的影响 The effects of miR-424 on the expression level of MMP-2 and MMP-9

### 2.4 荧光素酶活性检测miR-424对E2F6靶序列的作用

Targetscan分析得到E2F6上miR-424可能的靶向序列如图 4所示，将构建的pmirGLO/E2F6-3’UTR与pmirGLO/ E2F6-3’UTR mut荧光报告载体分别与miR-424共转染到A549细胞后，检测荧光素酶活性，结果如图 4所示，过表达miR-424后能够下调野生型E2F6 3UTR的报告载体的荧光素酶活性，而抑制miR-424的表达后野生型E2F6 3’UTR的报告载体的荧光素酶活性上调，说明miR-424对E2F6有靶向作用。而转染突变型E2F6 3’UTR的报告载体组的荧光素酶活性没有明显降低。实验结果暗示miR-424能与E2F6 3’UTR互补结合。

**4:**
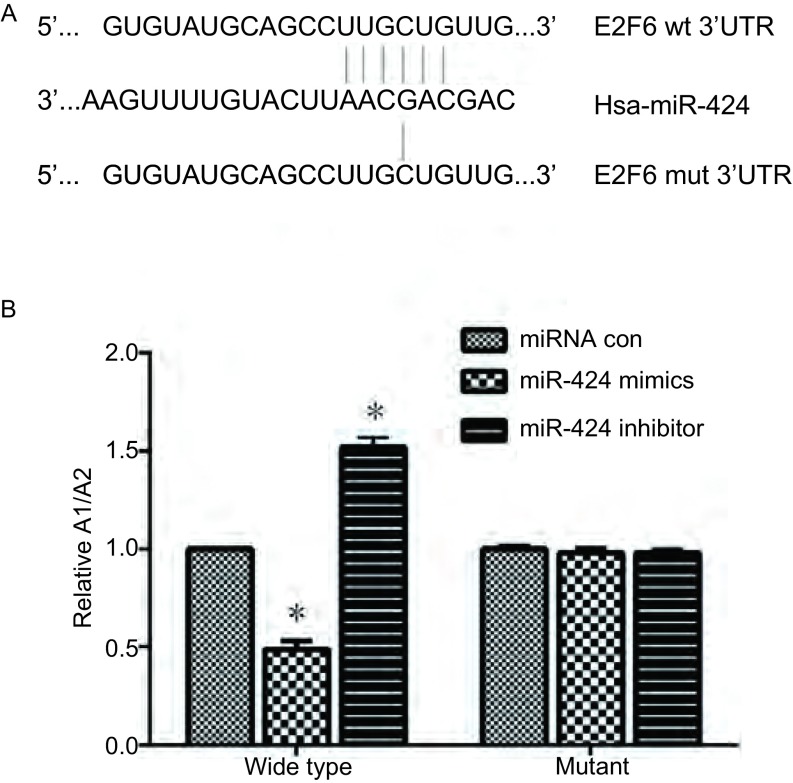
荧光酶素活性检测miR-424对E2F6靶序列的作用。A：Targetscan分析miR-424的靶向序列；B：miR-424对E2F6靶序列的作用。^*^：与对照组相比，*P* < 0.05。 The role of miR-424 to the target sequence of E2F6 by detecting enzymatic activity. A: Targeting sequence of miR-424 by Targetscan analysising; B: The role of miR-424 to the target sequence of E2F6. ^*^: compared with the control, *P* < 0.05.

### 2.5 miR-424负向调控NSCLC细胞中E2F6的表达

Western blot结果如图 5所示，与对照组相比，转染miR-424后，A549细胞中E2F6的表达明显下调，而转染miR-424 inhibitor组细胞中E2F6的表达明显上调。

**5:**
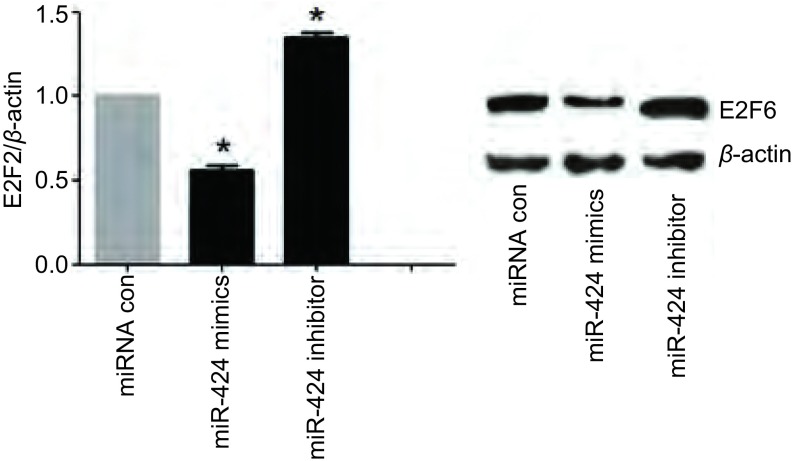
miR-424负向调控E2F6的表达。^*^：与对照组相比，*P* < 0.05。 miR-424 can negatively regulating E2F6 expression. ^*^: compared with the control, *P* < 0.05.

## 3 讨论

肺癌是目前全球范围内恶性肿瘤之一，NSCLC是最常见的肺癌类型，也是肺癌治疗的重点，研究表明，miRNA与多种癌症的发生发展密切相关^[7]^，且多种miRNA定位于基因组上与癌症相关的脆性位点^[8]^，说明其在癌症的发生过程中可能起着至关重要的作用。近年来大量的研究证实多种miRNA的异常表达与NSCLC密切相关^[9]^，如miR-138能够通过抑制PDK1而抑制NSCLC的增殖^[10]^，miR-21、miR-143及miR-181a的表达水平与NSCLC临床病理及预后密切相关^[11]^。

研究表明，miR-424在多种肿瘤中表达异常，其在不同肿瘤中发挥的作用也不同^[12, 13]^，如miR-424能够通过抑制ChK1的表达而抑制宫颈癌的发展，可作为宫颈癌的潜在预后指标和治疗靶点，且miR-424与宫颈癌、胰腺癌和前列腺癌等多种肿瘤的侵袭密切相关^[14]^。本研究中，在NSCLC细胞中过表达miR-424后，细胞的生长和侵袭能力降低，提示miR-424在NSCLC可能发挥抑癌的作用。本研究中，在NSCLC细胞中过表达miR-424后，细胞侵袭能力降低，且侵袭相关蛋白MMP-2和MMP-9蛋白表达降低，说明miR-424能够抑制NSCLC细胞的侵袭。

为进一步研究miR-424调控NSCLC生长侵袭的分子机制，本研究利用双荧光酶活性实验验证了其直接靶基因-核转录因子E2F6。研究^[15]^表明，E2F6能够抑制DNA损伤引起的细胞凋亡，E2F6在宫颈鳞状细胞癌中表达下调，其可能与宫颈癌的发生发展密切相关。由于miR-424可抑制肾癌细胞增殖，抑制宫颈癌细胞迁移和侵袭能力，所以miR-424与癌症细胞的发生呈正相关关系；而E2F6的下调又能导致宫颈癌的发生；所以推测miR-424与E2F6呈负相关关系。而Western blot结果表明miR-424负向调控NSCLC细胞中E2F6的表达，与推测相符。但是由于E2F6的相关报道较少，在后续研究中，我们将对E2F6在NSCLC中发挥的功能及机制进一步研究。

综上所述，本研究表明，miR-424能够抑制NSCLC的生长和侵袭，其下游靶基因为E2F6。在后续研究中将深入探讨具体作用机制，研究这些为探索miR-424在NSCLC中的作用机制以及参与的网络调控奠定基础，也为NSCLC的临床生物治疗提供新的治疗靶标。
